# Development of a Measurement System Using Infrared Spectroscopy-Attenuated Total Reflectance, Principal Component Analysis and Artificial Intelligence for the Safe Quantification of the Nucleating Agent Sorbitol in Food Packaging

**DOI:** 10.3390/foods13081200

**Published:** 2024-04-15

**Authors:** Joaquín Hernández-Fernández, Jose Martinez-Trespalacios, Edgar Marquez

**Affiliations:** 1 Chemistry Program, Department of Natural and Exact Sciences, San Pablo Campus, University of Cartagena, Cartagena 130015, Colombia; 2 Department of Natural and Exact Sciences, Universidad de la Costa, Barranquilla 080002, Colombia; 3 Chemical Engineering Program, School of Engineering, Universidad Tecnológica de Bolivar, Parque Industrial y Tecnológico Carlos Vélez Pombo, Km 1 Vía Turbaco, Turbaco 130001, Colombia; jmartinezt@utb.edu.co; 4 Facultad de Arquitectura e Ingeniería, Institución Universitaria Mayor de Cartagena, Cartagena 130015, Colombia; 5 Grupo de Investigaciones en Química Y Biología, Departamento de Química Y Biología, Facultad de Ciencias Básicas, Universidad del Norte, Barranquilla 081007, Colombia

**Keywords:** sorbitol, nucleating agent, infrared spectroscopy machine learning, RMSE, SVR

## Abstract

Sorbitol derivatives and other additives are commonly used in various products, such as packaging or food packaging, to improve their mechanical, physical, and optical properties. To accurately and precisely evaluate the efficacy of adding sorbitol-type nucleating agents to these articles, their quantitative determination is essential. This study systematically investigated the quantification of sorbitol-type nucleating agents in food packaging made from impact copolymers of polypropylene (PP) and polyethylene (PE) using attenuated total reflectance infrared spectroscopy (ATR-FTIR) together with analysis of principal components (PCA) and machine learning algorithms. The absorption spectra revealed characteristic bands corresponding to the C–O–C bond and hydroxyl groups attached to the cyclohexane ring of the molecular structure of sorbitol, providing crucial information for identifying and quantifying sorbitol derivatives. PCA analysis showed that with the selected FTIR spectrum range and only the first two components, 99.5% of the variance could be explained. The resulting score plot showed a clear pattern distinguishing different concentrations of the nucleating agent, affirming the predictability of concentrations based on an impact copolymer. The study then employed machine learning algorithms (NN, SVR) to establish prediction models, evaluating their quality using metrics such as RMSE, R^2^, and RMSECV. Hyperparameter optimization was performed, and SVR showed superior performance, achieving near-perfect predictions (R^2^ = 0.9999) with an RMSE of 0.100 for both calibration and prediction. The chosen SVR model features two hidden layers with 15 neurons each and uses the Adam algorithm, balanced precision, and computational efficiency. The innovative ATR-FTIR coupled SVR model presented a novel and rapid approach to accurately quantify sorbitol-type nucleating agents in polymer production processes for polymer research and in the analysis of nucleating agent derivatives. The analytical performance of this method surpassed traditional methods (PCR, NN).

## 1. Introduction

In 2019, world plastic production increased, reaching 360 million tons; half of these were attributed to disposable products, such as plastic bags made mainly of polyethylene (PE) [1,2,3]. Due to their excellent physicochemical properties and economic feasibility, synthetic plastics have been used in multiple industries, most frequently in the food industry as a packaging material [4,5,6]. Packaging is one of the most critical processes in food preservation because it preserves, protects, and provides essential information about the product while allowing its distribution and marketing [7,8]. Interactions between packaging and food can harm the desired product’s quality and safety. When foods are packaged in polymer-based materials, changes in aroma sorption and transfer of adverse flavors from the packaging to the food occur, leading to significant product deterioration [9,10].

In the food industry, using materials like copolymers and homopolymers, such as packaging materials or films, is gaining momentum. The mixture of ethylene/propylene copolymer [isotactic polypropylene (PP) and high-density polyethylene (HDPE)] is used as a packaging material to improve the processability of PP and its resistance to cracking and impact [11]. PP is highly crystalline, which gives it high resistance to rigidity. However, the low-temperature impact resistance of PP is sensitive to product testing, manufacturing, and end-use conditions, resulting in a loss of impact strength or toughness. To overcome this problem, use is made of random copolymers of PP with PE [11,12]. Polyolefins such as PE and PP have been increasingly used as packaging materials and containers to form sheets and films, among other things. However, since these tend to have little transparency, they fail to fully satisfy the demand for packaging for food containers that allow their contents to be seen from the outside [13].

**Figure 1:**
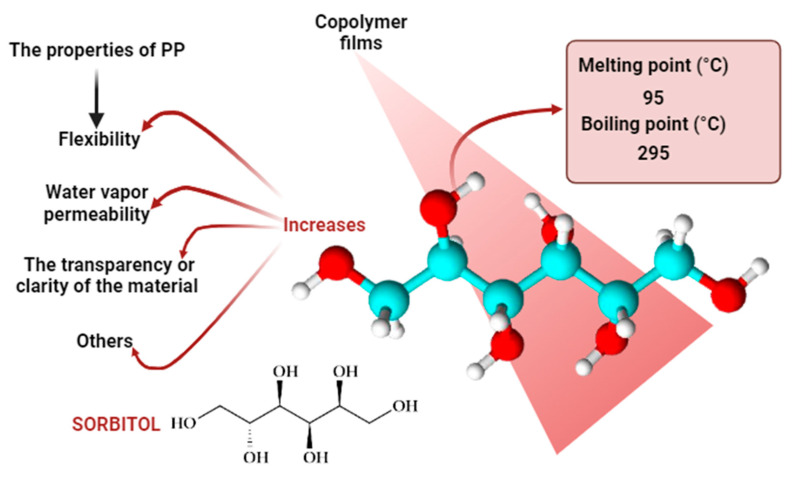
Improvements produced by sorbitol in food packaging.

At least four different crystal forms of PP have been identified: α-PP in a monoclinic structure, β-PP in a trigonal structure, γ-PP in an orthorhombic structure, and mesomorphic smectic forms [14]. The β crystal phase has garnered significant attention due to its outstanding properties, such as increased elongation before breaking, lower density, greater transparency, and enhanced impact and tear resistance, among others. The conventional method used to obtain the β crystal form involves introducing nucleating agents into the PP matrix [15]. During the production of food packaging, optimizing the physical properties of these containers, especially for industrial applications, is essential. To achieve this, nucleating agents are employed (Figure 1). Nucleating agents play a crucial role in altering the crystallization and morphology of semi-crystalline polymers, aiming to enhance various material properties such as optical transparency and mechanical performance [16,17]. Their effectiveness is closely linked to their ability to generate nuclei during the polymerization process. It has been observed in different systems that the activity of nucleating agents varies with the crystallization temperature [18,19,20] and/or the cooling rate.

The capability of sorbitol-derived compounds to efficiently induce crystallization in the α form of isotactic polypropylene (i-PP) is widely recognized and has been the subject of detailed investigations [21,22,23,24,25,26]. Even in small quantities, these nucleating agents have the power to significantly enhance transparency and reduce turbidity in products manufactured with this polymer, earning them the designation of “clarifiers.” Unlike many other nucleating agents, sorbitol derivatives are designed to dissolve and recrystallize in the molten polymer, facilitating the formation of an extensive and well-distributed three-dimensional nanofibrillar network [14,27]. Additionally, sorbitol is considered non-toxic and can be used in materials intended for food contact, making it a suitable choice for the manufacturing of food packaging materials [28].

**Figure 2:**
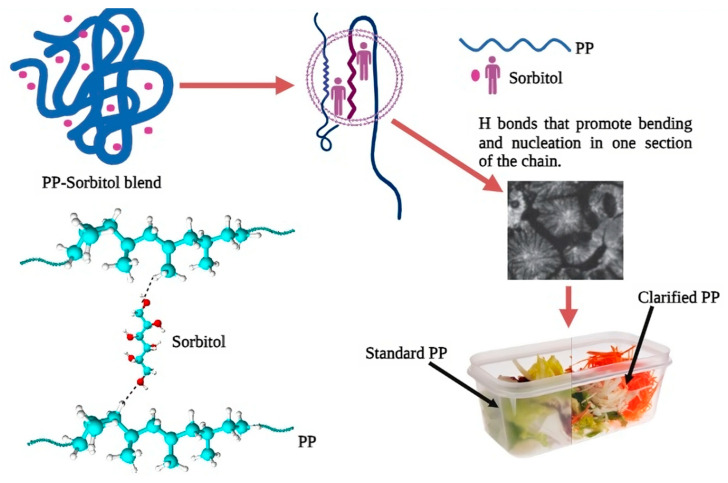
Function of sorbitol as a nucleation agent or clarifying agent for food packaging. The photo of the packaging was taken from the Millilliken page.

When adding nucleating agents to the polymer matrix, at least three aspects should be taken into consideration. Firstly, it is essential to assess the thermodynamic compatibility within the nucleating agent–polymer system. Conventional nucleating agents are insoluble in PP, whereas sorbitol-based nucleating agents may dissolve depending on their concentration during the process. Secondly, self-nucleation occurs during the crystallization of a semi-crystalline polymer, even without the presence of nucleating agents. The purpose of introducing nucleating agents is to enhance nucleation efficiency, resulting in smaller spherulite sizes (Figure 2). Nucleation efficiency increases linearly within a specific concentration range of the nucleating agent. Even if it is possible to induce PP to crystallize into β crystals, adding an excessive amount beyond that range does not contribute to improving nucleation efficiency. Lastly, the dispersion of nucleating agents within the PP matrix is of great importance as it directly impacts nucleation efficiency. Increasing the amount of nucleating agents negatively affects their dispersion within the PP matrix. Therefore, according to the literature [25,29,30], the quantity of nucleating agents added should not exceed 1% by weight.

Currently, nucleating clarifying agents (NCAs) have been widely used in the production of PP. The most common sorbitol-based NCAs are marketed under brands such as Millad, Irgaclear, and Irgaclear DM. These additives can enhance the physical properties and optical clarity while aiding in the PP production process [31]. Some of these NCAs, such as dibenzylidene sorbitol (DBS, FCM No. 674), are listed as positive additives by the European Commission (EU). These additives are not subject to specific migration limits (SML), which means they can be used freely in the manufacturing of plastics intended for food contact without particular restrictions. The only NCA with a set specific migration limit of 5 mg kg^−1^ is bis(4-propylbenzylidene) propylsorbitol (PBPS, FCM No. 808) [32].

Verifying the levels of polyols in food is crucial for quality control and nutritional information. This control is necessary and vital for consumer health, as significant amounts of polyols can lead to intolerance symptoms such as abdominal distension, laxative effects, abdominal discomfort, and flatulence [33].

Sorbitol-based NCAs in FCM have received little attention in the scientific literature [34,35], and although there is extensive research on the physical properties of these NCAs, their quantification and detection have posed many challenges due to their high boiling point, low volatility, and lack of chromophores [36]. In addition, the analysis of FCM additives needs to be improved by the need for commercial analytical standards of proven purity [37,38]. These problems have been solved with the use of analytical methods such as high-performance liquid chromatography (HPLC) [39,40,41] and gas chromatography (GC) [42,43]. An example is the case of FCM No. 674, with which the silylation technique was used to analyze GC. Still, no quantitative data were provided. Despite being one of the most used NCAs in the production of PP to improve its physical properties and make it an attractive choice against competition like polystyrene, there are very little published analytical data on its identification in FCM [32]. Mid-infrared spectrometry is usually used to identify functional groups of organic molecules. Its use in quantitative analysis has been increasing in the last two decades, thanks to the popularity of the Fourier transform (FTIR) technique. It provides a fast measurement of the spectrum and the signal/noise ratio advantageously compared to other instruments [44]. This technique has been used to quantify samples of pesticides [45,46,47,48,49], food [50,51,52], cosmetics [53,54], pharmaceutical products, and their main active ingredients [55,56,57,58,59], among others. However, just as with analytical techniques such as GC, there are very few works on the quantification of sorbitol and its derivatives by this technique.

In laboratories like the NICC, attenuated total reflectance Fourier transform infrared spectrometry (ATR-FTIR) is initially employed as a preliminary assessment before conducting more complex and costly analyses, such as gas chromatography-mass spectrometry (GC-MS) and gas chromatography with flame ionization detection (GC-FID), to identify and quantify substances. ATR-FTIR is fast and cost-effective, requiring minimal sample preparation, such as simple powder homogenization, and can be used in a portable device [60]. To identify unknown substances using ATR-FTIR, spectral libraries are used to compare the spectrum of the unknown substance with the spectra of known substances. The success of identification largely depends on the number of substances registered in the libraries. While matching spectra of pure substances to the library is easy, it becomes more challenging when dealing with mixtures containing multiple components. Even commercially available mixture search algorithms have limitations in practical utility [60].

In recent years, the food industry has been studying and developing methodologies for the detection of food packaging deterioration [61,62] based on Fourier transform infrared spectroscopy (FTIR), as it holds excellent potential as a foundation for quantitative methodologies [63]. FTIR is advantageous because it is non-invasive, non-destructive, allows real-time analysis, is relatively cost-effective, requires small sample quantities, and has straightforward sample preparation [64,65]. To overcome the limitations of ATR-FTIR, it can be combined with chemometric data processing, a powerful tool for extracting relevant information from high-dimensional data (such as spectra), enabling both qualitative and quantitative analyses. Currently, chemometric methods combined with FTIR spectroscopy are being developed to study food deterioration, employing techniques like principal component analysis (PCA), partial least squares regression (PLS-R), nearest neighbors, and neural networks, among others. These methods utilize mathematical and statistical approaches to select the best treatment of analytical data and experimental procedures [63]. Thanks to these multiparametric kinetic studies, progress has been made in interpreting and understanding complex reactions related to food quality. Machine learning plays a pivotal role in enhancing processes in the food industry by enabling the creation of models that forecast the best solution for specific data. This field encompasses both supervised and unsupervised learning, data preprocessing, feature engineering, model selection, evaluation, and optimization techniques. These methodologies are employed to address various challenges associated with optimizing food production. There is a growing trend in the food industry to leverage machine learning to enhance manufacturing efficiency, reduce waste, and tailor customer experiences. It is also used for early detection of food safety hazards, such as contaminants or spoiled products before they reach consumers [66,67,68].

Anticipated is a surge in the application of machine learning in the food sector as more companies recognize its potential to enhance customer satisfaction and boost productivity. Additionally, this technology can be harnessed to improve nanotechnological operations and optimize the preservation of fruits and vegetables. Through machine learning algorithms, it becomes possible to identify patterns related to numerous factors that influence the quality of preserved products by analyzing historical data. It can also be used to determine the optimal combinations of parameters that maximize product longevity [69].

Given the limited number of studies on the quantification of sorbitol in food packaging and the absence of studies focused on the application of ATR-FTIR spectroscopy and machine learning as tools for quantifying these additives, the main objective of this study is to develop a fast, selective, and accurate method for the direct determination and quantification of a sorbitol-type nucleating agent present in food packaging made from impact copolymers (PP and PE) using spectroscopy. This approach is based on attenuated total reflectance Fourier transform infrared technology (ATR-FTIR) and is complemented by principal component analysis and artificial intelligence. The combined application of these tools not only enables the precise determination of appropriate sorbitol amounts in food packaging but also facilitates real-time monitoring of sorbitol dosage during industrial packaging production processes, even in high-pressure situations. Moreover, it contributes to minimizing operational errors associated with traditional sorbitol analysis in industrial environments and reducing response times in industrial process controls, ultimately enhancing process safety. This innovative approach aims to address the existing research gap by providing an advanced and efficient solution for sorbitol quantification in industrial food packaging applications.

## 2. Materials and Methods

### 2.1. Materials

Copolymer blends of propylene and polyethylene were evaluated at concentrations ranging from 0.57% to 53.9% polyethylene. Ethylene and propylene monomers were supplied by Esenttia. Cyclohexane (≥99%) with ACS Reagent grade was obtained from Sigma Aldrich, and Sorbitol Parteck^®^ (182.17 g/mol) was provided by Merck KGaA, Darmstadt, Germany, as part of their EMPROVE^®^ ESSENTIAL product line.

The polymerization process consisted of three stages: pre-polymerization of propylene in suspension, homopolymerization of ethylene in suspension, and copolymerization of propylene-ethylene in the gas phase. Pre-polymerization was carried out in a 2L stainless steel reactor at 60 °C, using TEAL as a cocatalyst. Approximately 50 mg of ZN catalyst was added, and the solution was saturated with propylene at 0.1 MPa. Ethylene homopolymerization was initiated after 20 min with the injection of ethylene at 0.6 MPa, continuing for 30 min with continuous feed. During copolymerization, a continuous mixture of ethylene/propylene was introduced at 0.8 MPa from the bottom of the reactor, while the unreacted monomer mixture was simultaneously discharged through the reactor drain [70]. Combinations of polypropylene and ethylene were mixed for 7 min at 25 °C using a standard Prodex Henschel 115JSS mixer (Hainesport, NJ, USA) at 800 rpm. Subsequently, the samples were closely joined using a Welex-200 24.1 extruder (KD Capital Equipment, LLC.; California City, CA, USA) and melt extrusion. Temperatures inside the extruder were recorded at specific levels: 190 °C, 195 °C, 200 °C, 210 °C, and 220 °C. Next, the mixtures were compressed and molded into films using a hot press, CARVER 3895, with dimensions of 300 mm in diameter and 100 µm in thickness. The small pellets obtained with different amounts of ethylene were placed between two metal plates pressed together. Heat was applied until a thin film was obtained. This process is delicate and requires time to avoid material deterioration due to heat.

### 2.2. Preparation of Copolymers Samples with Varying Concentrations of Sorbitol

To create copolymers samples with sorbitol, the following procedure was conducted: individual weights of 0.0, 0.5, 1, 1.4, 1.8, 2, 2.5, 3.75, and 4 g of sorbitol were measured. Each amount of sorbitol was mixed with 1 kg of virgin copolymer resin. Subsequently, each mixture was pre-blended at 800 rpm for 7 min using a standard Prodex Henschel 115JSS mixer. Following this, each sample was extruded in a Welex-200 24 extruder. This extruder operates with five temperature zones along the entire extrusion process, maintaining temperatures of 190, 195, 200, 210, and 220 °C. This ensures a uniform mixture. At the end of the extruder, a molten PP-Sorbitol blend is obtained. For each type of molten blend, 30 g of molten material was fed into a CARVER 3895 hot press. In this CARVER machine, the samples were compressed to create films with a diameter of 300 mm and a thickness of approximately 100 µm. The films obtained in the experiment were identified as PP/PE1 (0 ppm sorbitol), PP/PE2 (500 ppm sorbitol), PP/PE3 (1000 ppm sorbitol), PP/PE4 (1400 ppm sorbitol), PP/PE5 (1800 ppm sorbitol), PP/PE6 (2000 ppm sorbitol), PP/PE7 (2500 ppm sorbitol), PP/PE8 (3750 ppm sorbitol), and PP/PE9 (4000 ppm sorbitol).

#### Extraction of Sorbitol from Copolymer Samples

Samples of copolymer in different forms (films, pellets, and ground material) to which sorbitol was added were obtained. Cyclohexane was used as the extraction solvent, and the ultrasonication technique was employed (in a conventional laboratory ultrasonic bath). For the ultrasonic bath, 5 g of PP was added to a 30 mL vial, and then 20.0 mL of the internal standard solution was added using a 5.0 mL micropipette. The sonication process was carried out for three hours in the ultrasonic bath, maintaining the temperature under control, with a maximum of 50 °C. After the sonication process was completed, the vials were removed from the ultrasonic bath and left to stand for 10 min. Disposable PTFE syringe filters were used to filter the extracted sorbitol sample solutions. In the case of crushed, pelleted, and films, the extraction took place for 60 min in the ultrasonic bath, with the solution being agitated for at least 25 s every 10 min.

### 2.3. Acquisition of Spectra

**Figure 3:**
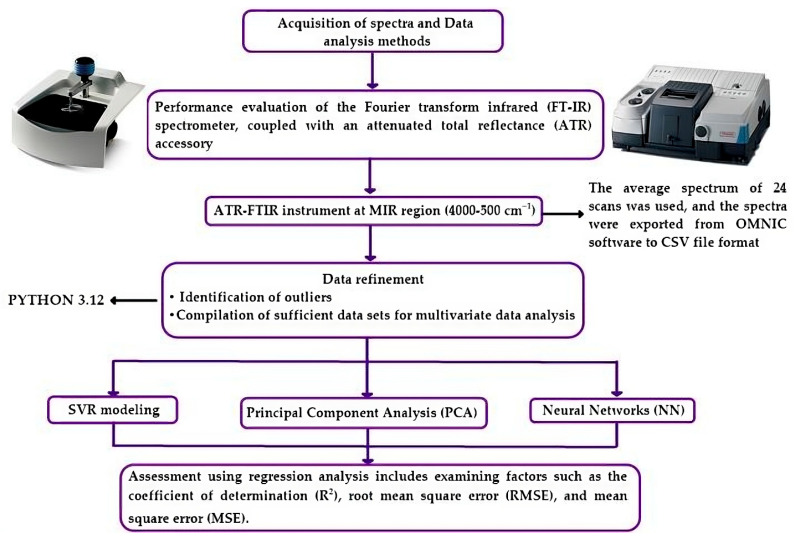
Experimental design for identification and quantification of sorbitol in food packaging.

Before the analysis, a performance evaluation of the Fourier transform infrared spectrometer (FT-IR) was conducted, along with an attenuated total reflectance (ATR) accessory (Nicolet 6700 FT-IR spectrometer with Smart iTR™ attenuated total reflectance accessory: Thermo Fisher Scientific Co., central, Waltham, MA, USA). At the beginning of the measurements, a background spectrum scan was performed using an empty diamond ATR cell in the range of 4000 to 500 cm^−1^, with 24 scans and a nominal resolution of 4 cm^−1^. The spectra of all samples were analyzed directly in the ATR cell. To obtain the actual sample spectrum, the background spectrum was subtracted from the sample spectrum. After each sample analysis, the ATR cell was meticulously cleaned with distilled water and ethanol, and an additional background scan was conducted to ensure its proper functioning. The FTIR spectral data were collected in the mid-infrared (MIR) region (4000–500 cm^−1^). The average spectrum of 24 scans was used, and the spectra were exported from the OMNIC™ Professional 7 with advanced ATR correction to CSV file format. Subsequently, they underwent chemometric analysis using PYTHON 3.12 (See Figure 3).

The use of Python as a programming language is gaining recognition as a valuable tool for data analysis in various fields. Despite recent advances in instrumental analysis that require robust and reliable data analysis, Python’s application in the field of analytical chemistry has been relatively unexplored. To address this challenge, multivariate analysis, or chemometrics, has been extensively applied to various types of data obtained through instrumental analysis. This study assesses the potential of Python for chemometrics and related areas in chemistry. In Python’s machine learning (ML) library, scikit-learn, many practical tools for chemometrics, such as principal component analysis (PCA), partial least squares (PLS), support vector machine (SVM), among others, are included. Additionally, other useful libraries are available on GitHub, such as pyMCR for multivariate curve resolution (MCR), 2Dpy for two-dimensional correlation spectroscopy (2D-COS), and more. These resources make it easy to create a computational environment for chemometrics using Python [71].

#### Smoothing of Spectra

As previously explained, spectral data derived from spectroscopy contain both the desired signal and a portion of noise. Generally, it is recognized that this noise component has a high-frequency spectral component. To address this issue, the best approach is to perform spectral smoothing [72,73]. Various smoothing methods have been documented in the literature, such as the moving average filter, Savitzky–Golay filter, and median filter, among others. In this study, integrated functions were applied for moving average (MA) smoothing, Savitzky–Golay (SG) smoothing, and Fourier transform. For the moving average smoothing, a window size of 5 was used, and for Savitzky–Golay smoothing, a polynomial degree of 2 and a window size of 5 were employed. Automatic smoothing was applied as an option in the spectrometer software (OMNIC 8.3). Although precise information about the specific algorithm used in this automatic smoothing method could not be obtained, it is important to note that this technique smoothens all parts close to the baseline without affecting spectral bands [74]

### 2.4. Data Analysis Methods

#### 2.4.1. Principal Component Analysis

Principal component analysis (PCA) is a multivariate statistical method used to investigate the correlation among various variables. Its purpose is to unveil the internal structure of these variables through specific principal components, essentially extracting key components from the original variables. PCA is one of the most commonly employed methods in the literature because it simplifies complex matrices and essential data for more comprehensible results.

In this research, PCA was employed to effectively extract information from spectral characteristics and eliminate redundant information in spectral data. The spectral data collected in the range of 4000 to 500 cm^−1^ underwent a smoothing process, followed by standardization of the spectra. PCA was then applied to analyze and verify differences among the samples. A calibration set was established, building a mathematical model between the FTIR spectra matrix and the concentration of the analytes studied in this investigation.

To develop and evaluate quantification models, a double cross-validation strategy was used with datasets containing only samples with the antioxidant. Various aspects were evaluated, including model accuracy, by determining errors like RMSEC, RMSECV, and RMSEP. Coefficients of determination (R^2^ for calibration, R^2^ for cross-validation, and R^2^ for validation) were calculated, and bias was also analyzed. These root mean square errors (RMSEC, RMSECV, and RMSEP) were calculated as described in Equation (1).

(1) $$\mathrm{RMSE}=\sqrt{\sum_{i=1}^{n}\frac{y_{i}-{\hat{y_{i}}}^{2}}{n}}$$

where:

$y_{i}$ represents the reference concentration of sample i;

$\hat{y_{i}}$ is the concentration of sorbitol predicted by the model;

n is the number of samples.

The predictive capacity of the model can be evaluated using the root mean square error of prediction (RMSEP); the smaller the RMSEP value obtained, the higher the degree of accuracy of the result obtained from the calibration. Additionally, the principal component regression (PCR) model was used to conveniently develop the calibration models for quantifying sorbitol in an impact copolymer.

#### 2.4.2. Definition of the Model with Neural Networks (NN)

In spectral data analysis, various regression approaches are employed, including techniques such as support vector machine (SVM) regression and artificial neural network (ANN) regression. Multiple linear regression (MLR) is another common method that establishes relationships between independent and dependent variables using a linear model. However, MLR has strict requirements, such as the constancy of residual variability, multivariate normality, and linearity, which can limit its applicability in certain cases.

On the other hand, SVM and ANN regression are frequently used for nonlinear prediction analysis in spectral data. For instance, ANN can capture highly nonlinear relationships between inputs and outputs, enabling the prediction of an output variable based on input data. While nonlinear models like SVM and ANN can deliver robust predictive performance in many applications, it is crucial to carefully address the issue of overfitting. Therefore, validation plays a pivotal role in nonlinear prediction analysis, ensuring the reliability and general applicability of models when applied to spectral data.

Neural networks are supervised learning algorithms that can be used as a regression model to predict values. It consists of simulating a network of neurons that are connected using some weights (wi); in turn, these neurons are classified into layers, with input layers (which would be the values of the predictors), hidden layers (the ones responsible for performing the activation functions) and finally the output layers (responsible for converting the data into the desired outputs). To improve the predictions of an NN model, activation functions are required to model very complex problems that are not only linear. In the same way, different network structures are usually tested to improve precision, varying the number of layers and several neurons.

#### 2.4.3. SVR Modeling

In this study, the SVR (support vector regression) was used with different hyperparameter conditions to train the dynamic system and subsequently obtain the appropriate model. The validation of the system was carried out, and it was compared with the PCR and NN methods. SVR training can be performed by Bayesian optimization or grid search, starting with estimating several sets of hyperparameters in case of the need for a better-starting set. The SVR will consider as efficient those results that minimize the loss function.

## 3. Results

### 3.1. Spectra Measured by FTIR

**Figure 4:**
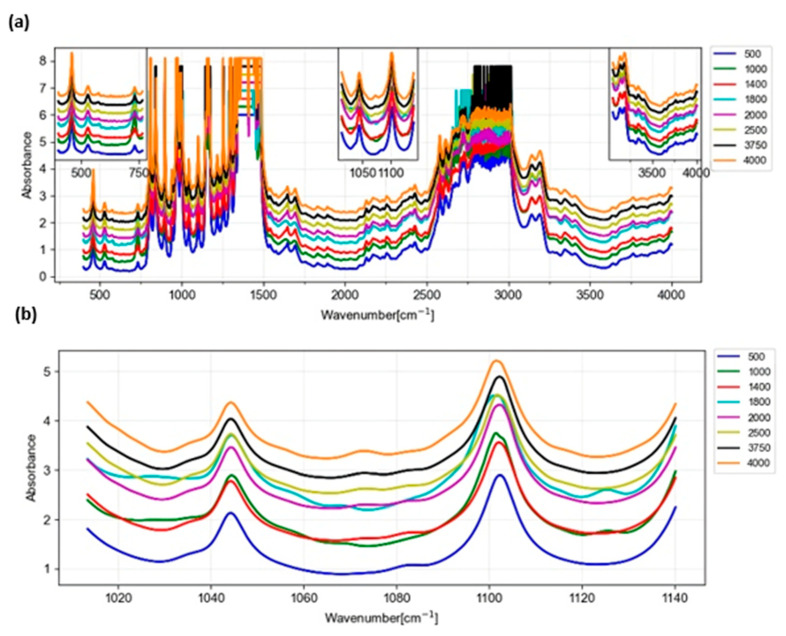
IR spectrum. (**a**) Complete spectrum. (**b**) Bands of the spectrum chosen for the analysis.

All the spectra in Figure 4b show a series of broad bands around 1045, 1140, and 1200 cm^−1^. These absorption bands in the region between 1050 and 1200 cm^−1^ correspond to the C–O–C bond and hydroxyl groups (–OH) linked to the cyclohexane ring of sorbitol. In addition to the absorption bands in the region between 3200 and 3500 cm^−1^, which correspond to the free –OH groups and bound to the cyclohexane of sorbitol, these bands are characteristic of sorbitol and are used to identify and quantify the presence of the sorbitol derivatives in samples [74,75,76].

### 3.2. Review of FTIR Data

**Figure 5:**
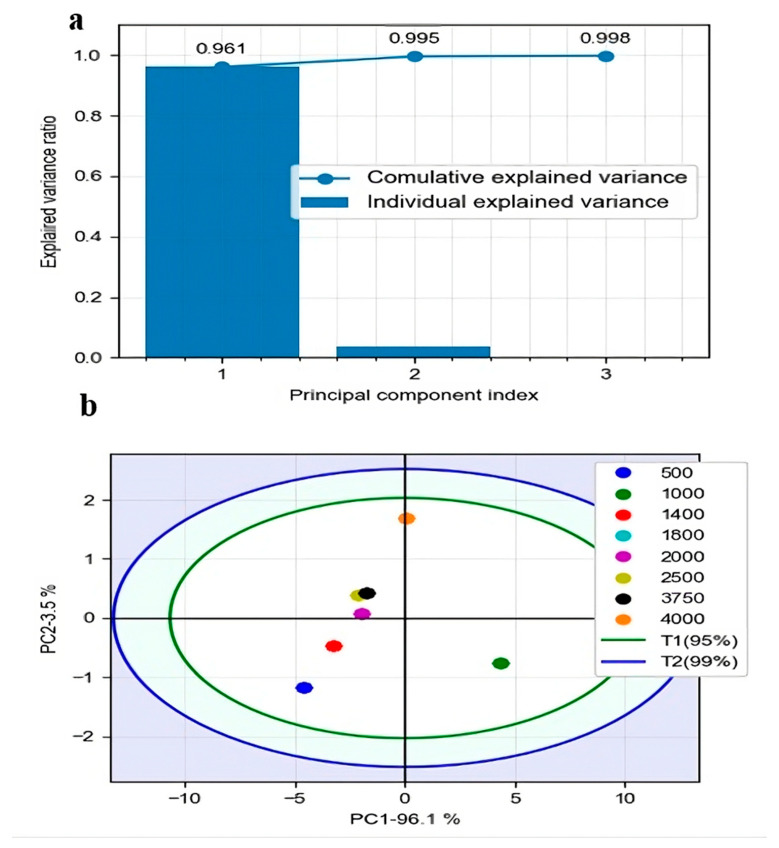
Principal component analysis (PCA) results. (**a**) Histogram of variance and cumulative variance explained by each principal component. (**b**) PCA score diagram.

The FTIR absorption spectra of the sample at different concentrations were obtained, followed by a smoothing and standardization process. Subsequently, PCA was applied to the data to define atypical samples and verify that the region of the spectrum analyzed provided sufficient information to characterize the different concentrations. In Figure 5a, it is possible to see that only with the first two components is it possible to explain 99.5% of the variance; also, in Figure 5b, it can be seen that in a score graph with these first two components, it is possible to highlight a pattern between the different concentrations, in which the second component, the y-axis, explains the difference between the concentrations. In such a way, when moving to a more excellent value on the y-axis, the concentration of the sorbitol-type nucleating agent increases. Likewise, in Figure 5b, it can be seen that the samples of the same concentration do not present appreciable deviations; they overlap almost perfectly, and all the samples analyzed are within the Hotelling ellipse with a confidence of 95%, which shows that the results have a high degree of reliability. It is possible to affirm that with the spectrum range chosen for the FTIR tests and with only the first components of a PCA, it is possible to predict the concentrations of the nucleating agent present in food packaging based on an impact copolymer. Therefore, the first component states were established to continue analyzing the different machine learning algorithms [70].

### 3.3. Quality of the RMSE Prediction Models

The quality of the prediction models was validated using the RMSE calibration mean square error, the regression coefficient (R^2^), and the cross-validation mean square error (RMSECV). Table 1 defines the hyperparameters used to carry out the cross-validation procedure. The best configuration was analyzed from the prediction errors obtained.

**Figure 6:**
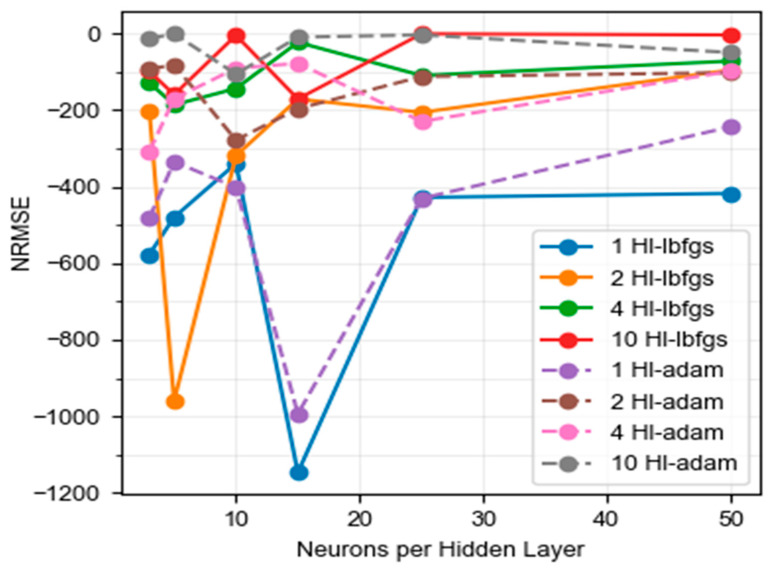
Result of cross-validation of the test score for NN.

High R^2^ and low values of RMSE indicate excellent precision and quality of the chosen models. The number of regression factors should be as few as possible to achieve a robust prediction model [77]. The value of these parameters proves that prediction models supported by spectral data are moderately accurate or punctual [64]. In Figure 6, it is possible to appreciate the evolution of the RMSE as the number of neurons per hidden layer increases; it is also possible to enjoy the behavior with different layers and the solver algorithms used. As expected, as the neural network architecture has more neurons, the solution generates fewer errors, which implies longer computation times. On the other hand, it is seen that architectures with a single hidden layer, regardless of the number of neurons, will generate results with high error rates. In general, the best behavior is achieved with Adam’s algorithm. The most balanced solution is reached with an architecture of two hidden layers, each with 15 neurons, using Adam’s algorithm. Since this solution achieved acceptable errors with better consumption of computational resources, it was the one used to obtain the predictions of this study.

### 3.4. Support Vector Machine (SVR)

**Figure 7:**
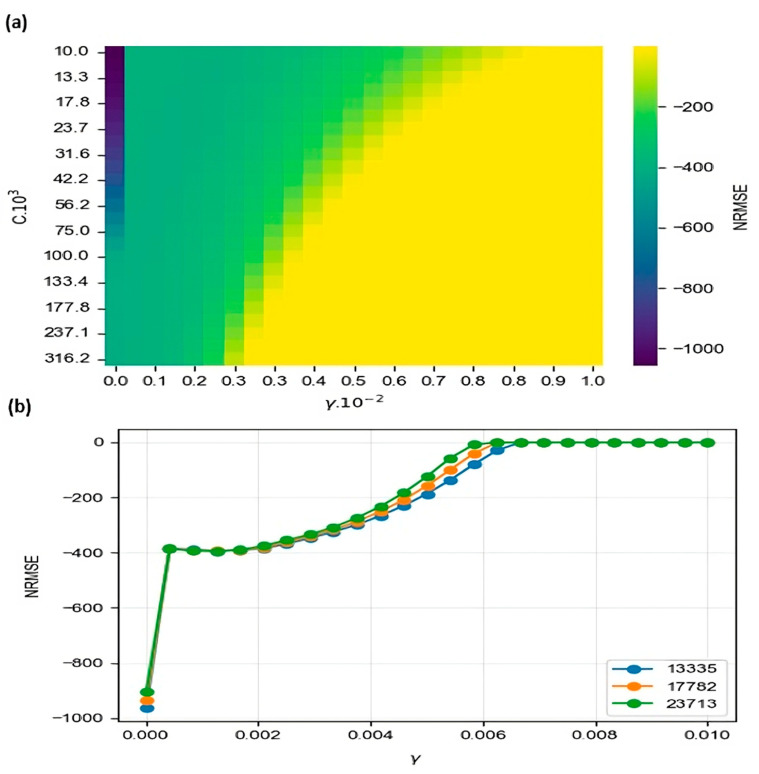
Test score cross-validation result for SVM. (**a**) All results. (**b**) Result for SVM for *C* = 13,335, 17,782, and 23,713.

This method converts the problem into a linear one with the help of transformations from the original space to higher-dimensional spaces. A specific error in the data was admitted, marked by the hyperparameter C, to avoid that when the error is minimized in the regression, it does not fall into overfitting [78]. In Figure 7, it is possible to appreciate the results achieved for the SVR algorithm. Initially, it was explored with a heat diagram for the two hyperparameters, analyzing the areas where it is possible to achieve lower NRMSE with the lowest possible C values (Figure 7a). Said zone was located for C values between 13,335 and 23,713. The most suitable γ value was analyzed in this zone (Figure 7b). The optimal values were chosen: C= 13,335 and γ = 0.007 [79].

### 3.5. Prediction Models

The validation results presented in this study demonstrated that PCA supported by the FTIR technique could accurately quantify the amount of the nucleating agent present in food packaging. Table 2 shows the comparison between the different algorithms (PCR, NN, and SVR) previously reported to estimate the quantities of the additive. It is possible to appreciate in Table 2 the errors reached from the selected hyperparameters with the best results for each technique used. It can be said that the PCR method has high errors in calibration and prediction. Still, it must be remembered that this is the simplest method and does not require hyperparameter calibration. On the other hand, with SVR, almost perfect predictions were obtained (R^2^ = 0.9999), both for calibration and prediction and with an RMSE with much lower values compared to the other two methods. Based on the analysis and comparison, it can be affirmed that the measurement method based on the ATR-FTIR coupled SVR model is novel, and to date, no bibliographic reports have been found for the quantification of this type of additive in food packaging [80,81,82].

**Figure 8:**
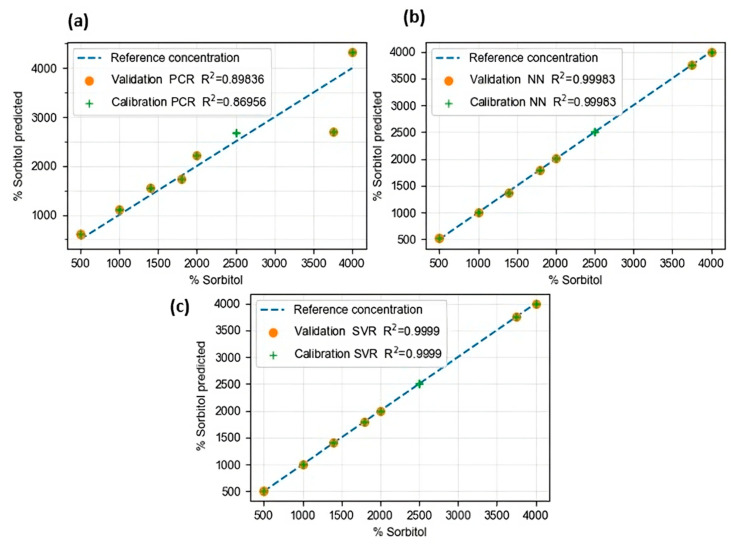
Real vs. prediction concentration of all ranges of Sorbitol samples by different machine learning algorithms. (**a**) Results for PCR, (**b**) results for NN, and (**c**) results for SVR.

In Figure 8, the predictions of the algorithms can be estimated both in the training samples and in the calibration samples. It can be highlighted that, indeed, the lowest results are achieved by PCR, and qualitatively, SVR and NN achieve very similar predictions.

## 4. Discussion

In this study, we address the critical need to quantify sorbitol-derived nucleating agents in food packaging materials (FCM), an area of research that has notably lacked attention in the scientific literature. While previous studies have explored the application of FTIR spectroscopy combined with chemometric techniques to assess unwanted substances in food [83], our research specifically focuses on the category of sorbitol-based nucleating agents.

The absence of commercial standards indicating the purity of these nucleating agents has been a significant challenge, complicating their analysis [36,84]. This is reflected in the limited analytical information on the nucleating agent FCM No. 674 despite its widespread use and known efficiency [79,85,86,87]. Our innovative methodology addresses this gap by using FTIR spectroscopy along with advanced machine learning techniques and principal component analysis (PCA).

Comparing our approach with other analytical techniques, such as LC-MS and GC-MS employed by some researchers, we highlight the efficiency and precision of our method in quantifying these nucleating agents in FCM. Additionally, we note the limitation of these techniques due to the lack of commercial analytical standards, emphasizing the innovation and valuable contribution of our study.

In contrast to previous studies that used techniques like GC-MS to address the low solubility of these nucleating agents, our FTIR-SVR methodology overcomes this limitation and achieves high precision in characterizing sorbitol concentrations in only two PCA components.

Tsochatzis’s study [32] provides valuable insight by using UHPLC-qTOF-MS, underscoring the importance of advances in analytical techniques for the accurate determination of nucleating agents regulated by the European Union [88]. However, our FTIR-SVR methodology offers an efficient and cost-effective alternative, proving to be accurate, fast, and suitable for compliance with food regulations.

In conclusion, our ATR-FTIR, coupled with the SVR approach, represents a significant contribution to the quantification of sorbitol-based nucleating agents in FCM. The combination of advanced spectroscopic techniques with machine learning has proven to be effective and precise, overcoming previous limitations and providing a valuable tool for laboratories seeking compliance with food legislation.

## 5. Conclusions

The results of the study demonstrate that the FTIR technique, coupled with PCA and SVR algorithms, is highly effective in accurately quantifying the sorbitol-type nucleating agent present in food packaging made from impact copolymers (PP and PE). The analysis of FTIR absorption spectra revealed characteristic bands around 1045, 1140, and 1200 cm^−1^, corresponding to the C–O–C bond and hydroxyl groups (–OH) linked to the cyclohexane ring of sorbitol. These absorption bands, along with those in the region between 3200 and 3500 cm^−1^, specific to free –OH groups and those bound to the cyclohexane of sorbitol, were crucial for identifying and quantifying sorbitol derivatives in samples.

The PCA analysis demonstrated that with only the first two components, 99.5% of the variance could be explained, and a clear pattern related to different concentrations of the nucleating agent was observed. The samples of the same concentration showed minimal deviations, confirming the reliability of the results within a 95% confidence level. The subsequent application of machine learning algorithms (NN, SVR) for prediction models involved hyperparameter optimization. The SVR algorithm, specifically, exhibited superior performance with an R^2^ value of 0.9999 and an RMSE of 0.100 for both calibration and prediction, outperforming PCR and NN. The chosen SVR model with two hidden layers, each with 15 neurons, and using Adam’s algorithm demonstrated the best balance between accuracy and computational efficiency. The validation results underscored the effectiveness of the ATR-FTIR coupled SVR model, with almost perfect predictions and a significant reduction in RMSE compared to other methods. The study contributes a novel approach for accurately quantifying sorbitol-type nucleating agents in food packaging, addressing a gap in existing research.

## Figures and Tables

**Table 1:** Chosen hyperparameter values.

Method	Hyperparameters	Values
NN	Neurons	[3; 5; 10; 15; 25; 50]
	Hidden Layers	[1; 2; 4; 10]
	Solver	[lbfgs; adam]
SVR	C	$10^{x}$, x ϵ [4; …; 5.5]
	Γ	[0; 0.07; …; 1] × 10^−2^

**Table 2:** Errors and R^2^ for the analyzed ML technique.

Algorithm	Calibration		Prediction	
	R^2^	RMSE	R^2^	RMSE
PCR	0.8695	407.80	0.8983	403.64
NN	0.9998	14.63	0.9998	16.40
SVR	0.9999	0.100	0.9999	0.100

## Data Availability

The original contributions presented in the study are included in the article, further inquiries can be directed to the corresponding authors.
